# *Giardia* Flagellar Motility Is Not Directly Required to Maintain Attachment to Surfaces

**DOI:** 10.1371/journal.ppat.1002167

**Published:** 2011-08-04

**Authors:** Susan A. House, David J. Richter, Jonathan K. Pham, Scott C. Dawson

**Affiliations:** Department of Microbiology, University of California Davis, Davis, California, United States of America; Washington University School of Medicine, United States of America

## Abstract

*Giardia* trophozoites attach to the intestinal microvilli (or inert surfaces) using an undefined “suction-based” mechanism, and remain attached during cell division to avoid peristalsis. Flagellar motility is a key factor in *Giardia*'s pathogenesis and colonization of the host small intestine. Specifically, the beating of the ventral flagella, one of four pairs of motile flagella, has been proposed to generate a hydrodynamic force that results in suction-based attachment via the adjacent ventral disc. We aimed to test this prevailing “hydrodynamic model” of attachment mediated by flagellar motility. We defined four distinct stages of attachment by assessing surface contacts of the trophozoite with the substrate during attachment using TIRF microscopy (TIRFM). The lateral crest of the ventral disc forms a continuous perimeter seal with the substrate, a cytological indication that trophozoites are fully attached. Using trophozoites with two types of molecularly engineered defects in flagellar beating, we determined that neither ventral flagellar beating, nor any flagellar beating, is necessary for the maintenance of attachment. Following a morpholino-based knockdown of PF16, a central pair protein, both the beating and morphology of flagella were defective, but trophozoites could still initiate proper surface contacts as seen using TIRFM and could maintain attachment in several biophysical assays. Trophozoites with impaired motility were able to attach as well as motile cells. We also generated a strain with defects in the ventral flagellar waveform by overexpressing a dominant negative form of alpha2-annexin::GFP (D122A, D275A). This dominant negative alpha2-annexin strain could initiate attachment and had only a slight decrease in the ability to withstand normal and shear forces. The time needed for attachment did increase in trophozoites with overall defective flagellar beating, however. Thus while not directly required for attachment, flagellar motility is important for positioning and orienting trophozoites prior to attachment. Drugs affecting flagellar motility may result in lower levels of attachment by indirectly limiting the number of parasites that can position the ventral disc properly against a surface and against peristaltic flow.

## Author Summary

*Giardia* is a widespread, single-celled, intestinal parasite that infects millions of people and animals each year. Colonization of the small intestine is a critical part of *Giardia*'s life cycle in any host. This colonization is initiated when cells attach to the intestinal wall via a specialized suction cup-like structure, the ventral disc. In the host, *Giardia* moves by beating four pairs of flagella; movement of the ventral pair has been implicated in attachment. This study shows that the beating of the flagella is not important for attachment, but rather for positioning *Giardia* close to the intestinal wall prior to attachment, and thus disproves the commonly held model of giardial attachment. This work implies that drugs targeting *Giardia* motility could prevent or slow attachment, leading to lower rates of infection.

## Introduction

Giardiasis is caused by acute or chronic infection with the single-celled, zoonotic parasite *Giardia intestinalis* [1]. Giardiasis is one of the most prevalent intestinal protozoal parasitic infections worldwide [2], resulting in several hundred million acute cases of malabsorptive diarrhea each year. The parasite persists in the environment as a dormant, infectious cyst [3], [4]. Infection of humans or other mammals is initiated by the ingestion of cysts from contaminated water or food [5]. Following ingestion, giardial cysts travel to the small intestine of the animal host, excyst and transform into the flagellated trophozoite. To avoid peristalsis and colonize the small intestine, trophozoites attach to the intestinal villi via a specialized microtubule structure, the ventral disc. The mechanism of attachment has been proposed to involve suction generated either by the ventral disc itself or by the regular beating of the ventral flagella [6], [7]. Both the molecular mechanism of attachment and the precise role of flagellar motility in attachment remain controversial.

**Figure 1:**
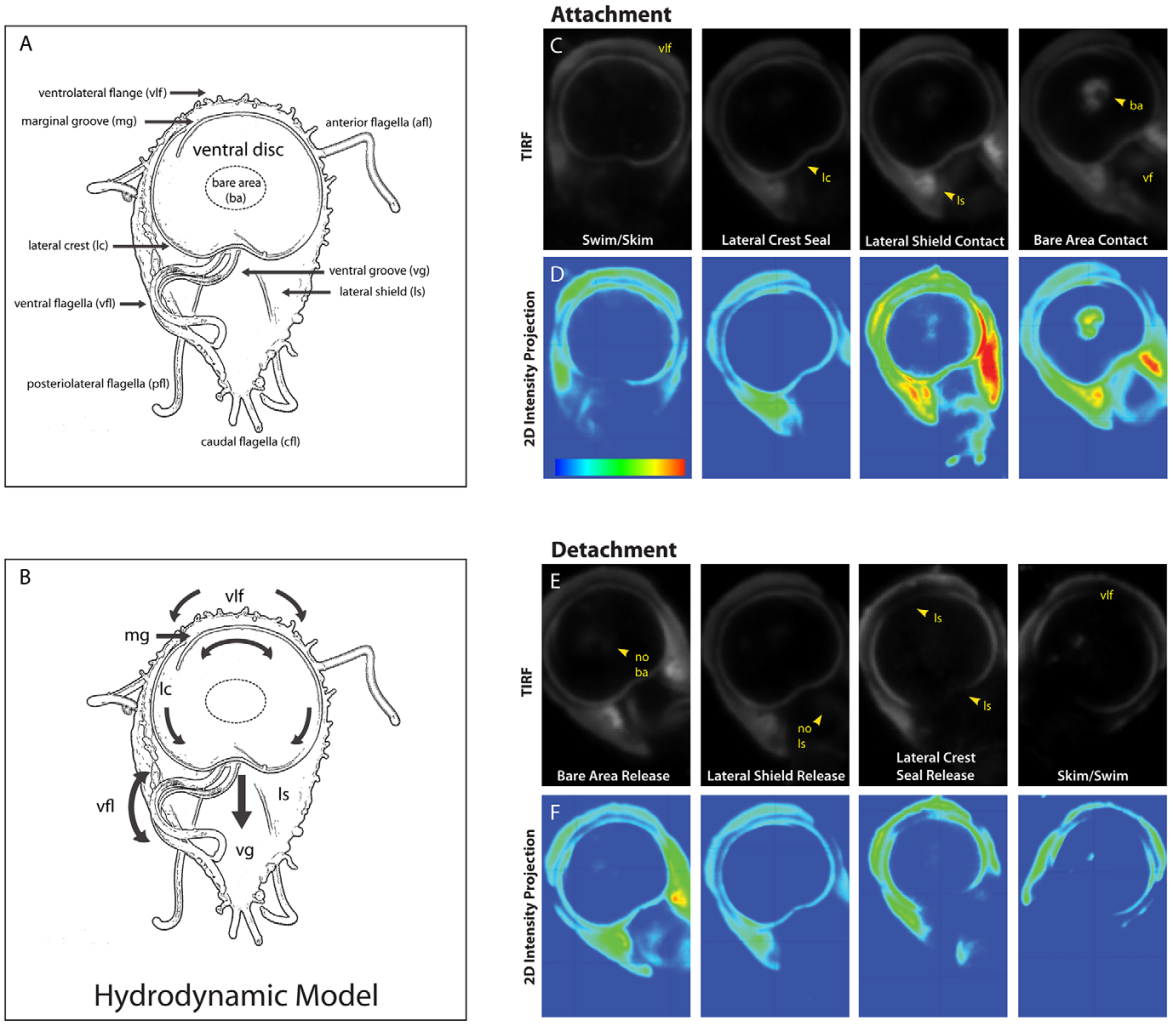
Sequence of the steps in surface contact during giardial attachment and detachment. The ventral surface of a trophozoite is shown in the panel (**A**) schematic, which highlights the four flagellar pairs (ventral flagella (vfl), caudal flagella (cfl), posteriolateral flagella (pfl), and the anterior flagella (afl) as well as critical cytological features including: the ventrolateral flange (vlf), the marginal groove (mg), the lateral crest (lc), the lateral shield (ls), and the ventral groove (vg). A schematic of the “hydrodynamic model” of attachment [7] including the currents of fluid (arrows) proposed to result from ventral flagellar beating, is shown in panel (**B**). According to this model, a negative pressure differential develops from fluid drawn under the ventrolateral flange, around the ventral disc (curved arrows), and into the ventral groove generating suction. Panel (**C**) shows the sequence of events that occur during the contact of the ventral disc and cell body within 100 nm of the coverslip surface. Panel (**D**) shows 2D intensity projections of the same images, indicating stronger contacts in “warmer” colors (red, yellow, green) and weaker contacts with “cooler” colors (indigo, blue). Note the contact of the ventrolateral flange (vlf) and the discontinuous contact of the disc periphery (lateral crest; lc) with the surface during skimming. Attachment is defined by the continuous disc seal (lateral crest), lateral shield (ls) pressure and lastly, bare area (ba) appearance. The posterior “tail” end of the trophozoite does not make contact with the substrate during attachment. Throughout attachment, the ventral flagella (vf) beat with the typical semi-sigmoidal beat pattern (see Video S1). Note the lack of visible anterior portals in the disc seal (for inflow) or breaks in this ventral disc perimeter seal during attachment (for proposed hydrodynamic outflow). In panels (**E**) and (**F** )detachment was monitored for the same cell, and the steps of trophozoite contact with the surface occur in reverse order (n = 97).

Trophozoites are bilaterally symmetrical with a flattened teardrop shape (∼15 µm long by 5 µm wide and 5 µm thick) and possess a complex microtubule cytoskeleton that includes eight flagella [8]. *Giardia's* flagella generate complex movements essential for motility, cell division, and access to suitable sites for attachment on the intestinal villi [9], [10]. The eight flagella are organized as four pairs: the anterior, the caudal, the posteriolateral and the ventral flagella (Figure 1A). *Giardia* axonemes possess long cytoplasmic regions that exit the cell body as membrane-bound flagella. All eight flagella have a canonical motile structure consisting of nine outer doublet microtubules surrounding the central microtubule pair, radial spokes and dynein arms [11]. While the role of flagellar motility in attachment remains speculative [9], the coordinated and differential beating of *Giardia's* eight motile flagella are known to be critical to cellular motility and division, and are possibly involved in encystation/excystation or chemotactic sensing [12].

The most widely held model of giardial attachment, the “hydrodynamic model” [7], [13], contends that flagellar motility is necessary for the initiation and maintenance of giardial attachment to surfaces. Specifically, the ventral flagella were proposed to produce a hydrodynamic current generating a suction pressure under the adjacent ventral disc. The model postulates that surrounding fluid is drawn through presumptive channels that initiate at the ventrolateral flange, flows under the marginal groove and lateral crest at the perimeter of the disc, and eventually exits at a channel in the posterior lip of the disc into the ventral groove, where the ventral flagella were thought to exit from the cell body (see Figure 1B). Cytological evidence has not corroborated the existence of these channels; thus, support for the “hydrodynamic model” has remained strictly observational or theoretical [7], [13]. Prior investigations have not distinguished between ventral flagellar beating that causes attachment and flagellar beating that merely coincides with attachment.

**Figure 2:**
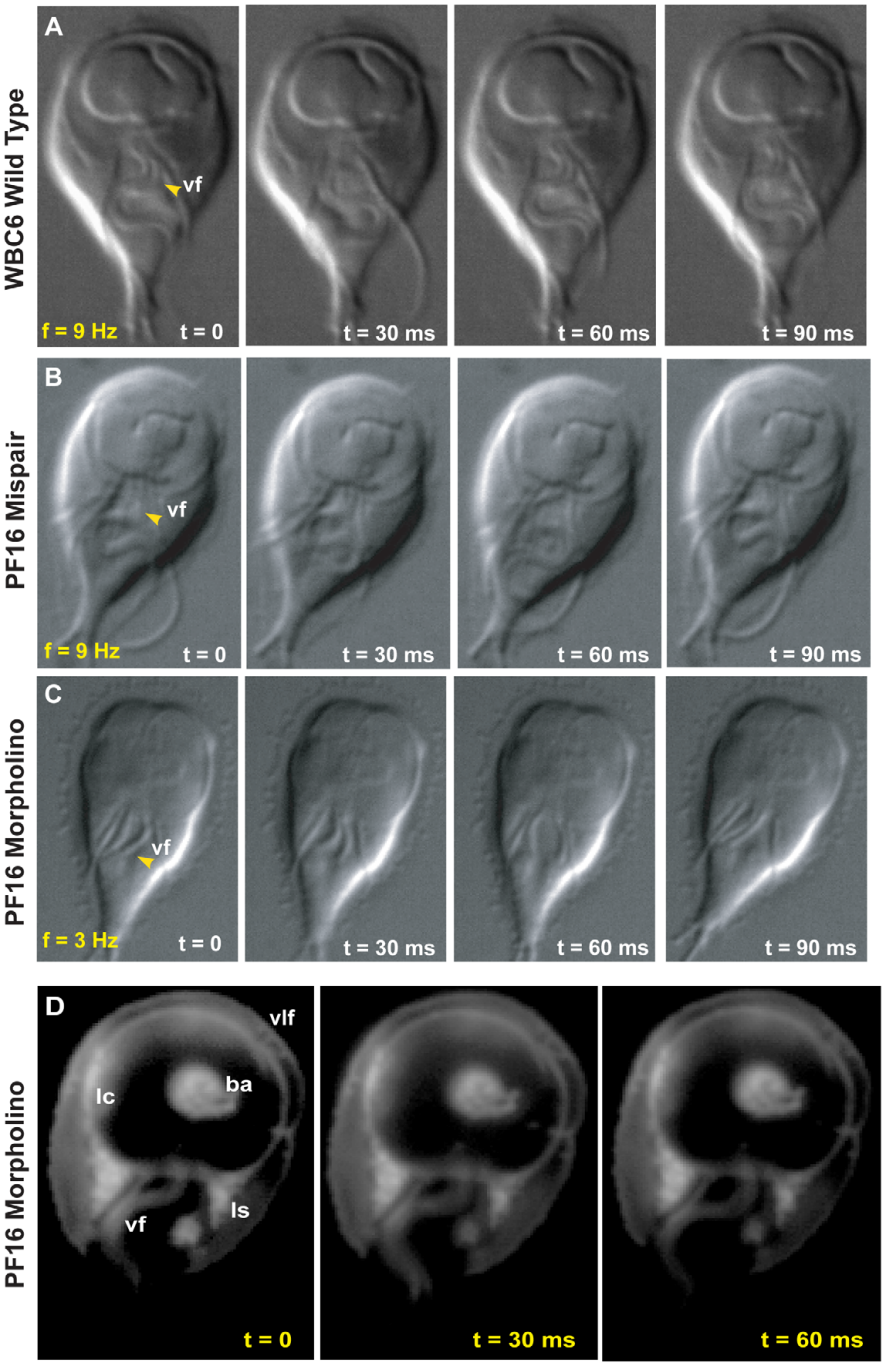
Morpholino knockdown of PF16 significantly reduces flagellar beat frequency but only mildly affects the ability to maintain attachment. Flagellar motility and morphology are shown using DIC for (**A**) wild type, (**B**) PF16 mispair morpholino and (**C**) the anti-PF16 morpholino. Both the wild type and the PF16 mispair control possess sigmoidal flagellar waveforms with similar frequencies (9 Hz), amplitudes and lengths (see also Video S2). In contrast, 24 hours after the electroporation of an anti-PF16 morpholino, ventral flagellar beating is slow and erratic with consistent pauses (n = 50, see also Video S4). At 48 hours, both the caudal and ventral flagella are shorter than in the wild type or the mispair morpholino control. Panel (**D**) shows trophozoite-surface contacts using cell membrane stained trophozoites with TIRFM (as in Figure 1). Note the continuous disc seal (lc), lateral shield (ls) and bare area (ba) contacts with the surface of the PF16 morpholino knockdown, despite erratic and transiently paralyzed ventral flagella (Video S4).

**Figure 3:**
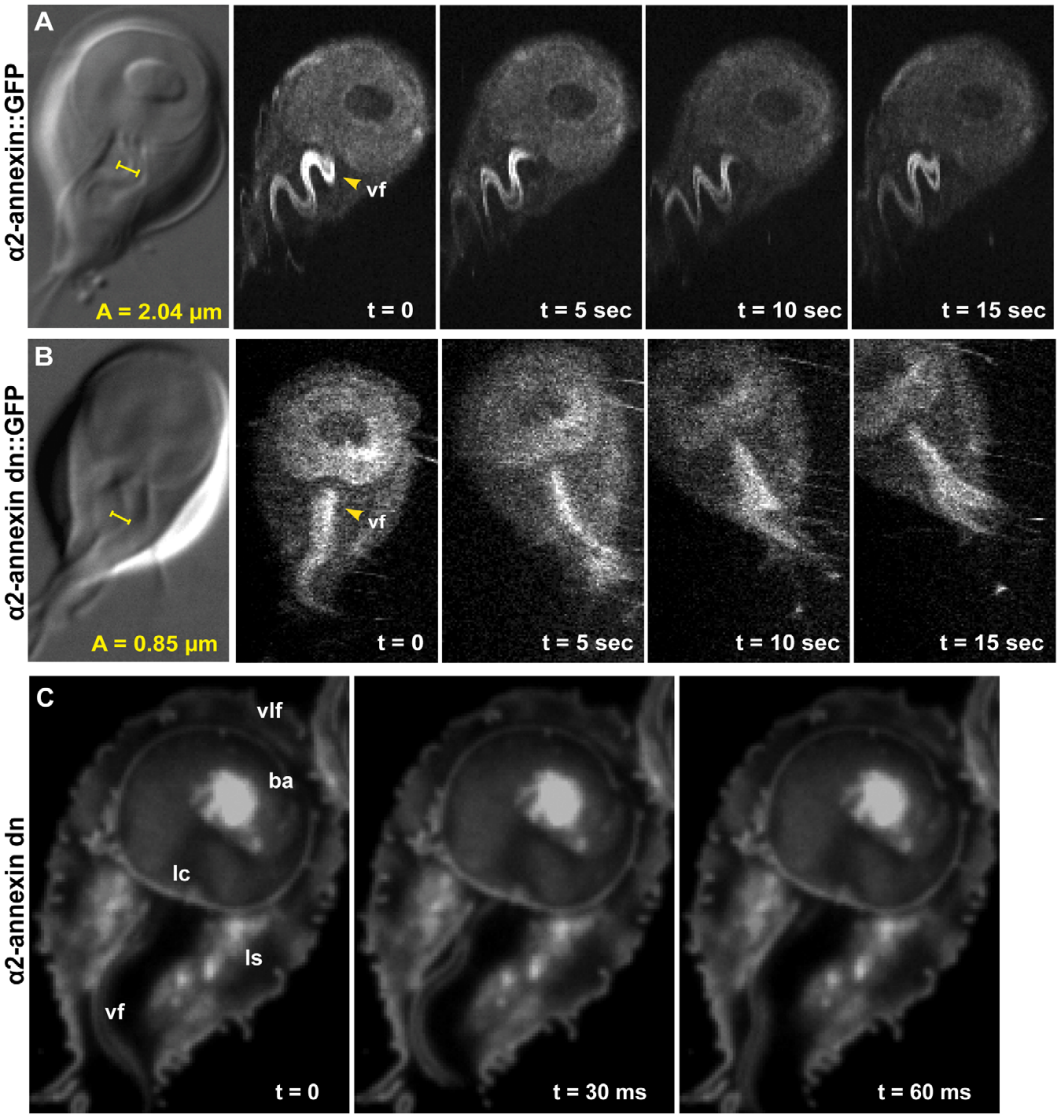
Overexpression of a dominant negative alpha2-annexin::GFP (D122A, D275A) decreases ventral flagellar waveform amplitude with only slight decreases in attachment. The role of the ventral flagella in attachment was determined using overexpression of a dominant negative form of the ventral flagella-associated alpha2-annexin (see Methods). DIC microscopy was used to measure the amplitude (A) of the ventral flagellar waveform (vf). Fluorescence microscopy was used to determine the localization of the GFP-tagged alpha2-annexin. Panel (**A**) shows that alpha2-annexin localizes primarily to the ventral flagella and diffusely to the plasma membrane of the ventral disc, yet GFP-tagging does not affect flagellar beat frequency or amplitude. (**B**) Overexpression of the dominant negative alpha2-annexin (D122A, D275A), or alpha2-annexin dn, results in a ventral flagellar (vf) beat with significantly decreased amplitude (n = 25; see also Video S3). Panel (**C**) shows trophozoite surface contacts using cell membrane stained trophozoites using TIRFM. As compared to the standard in Figure 1, proper substrate surface contacts are made, including a continuous disc seal (lc), lateral shield (ls) and bare area (ba). The same trophozoite surface contacts and disc perimeter seal are visible despite a diminished flagellar waveform amplitude and asynchrony between the flagella (see also Video S4).

**Figure 4:**
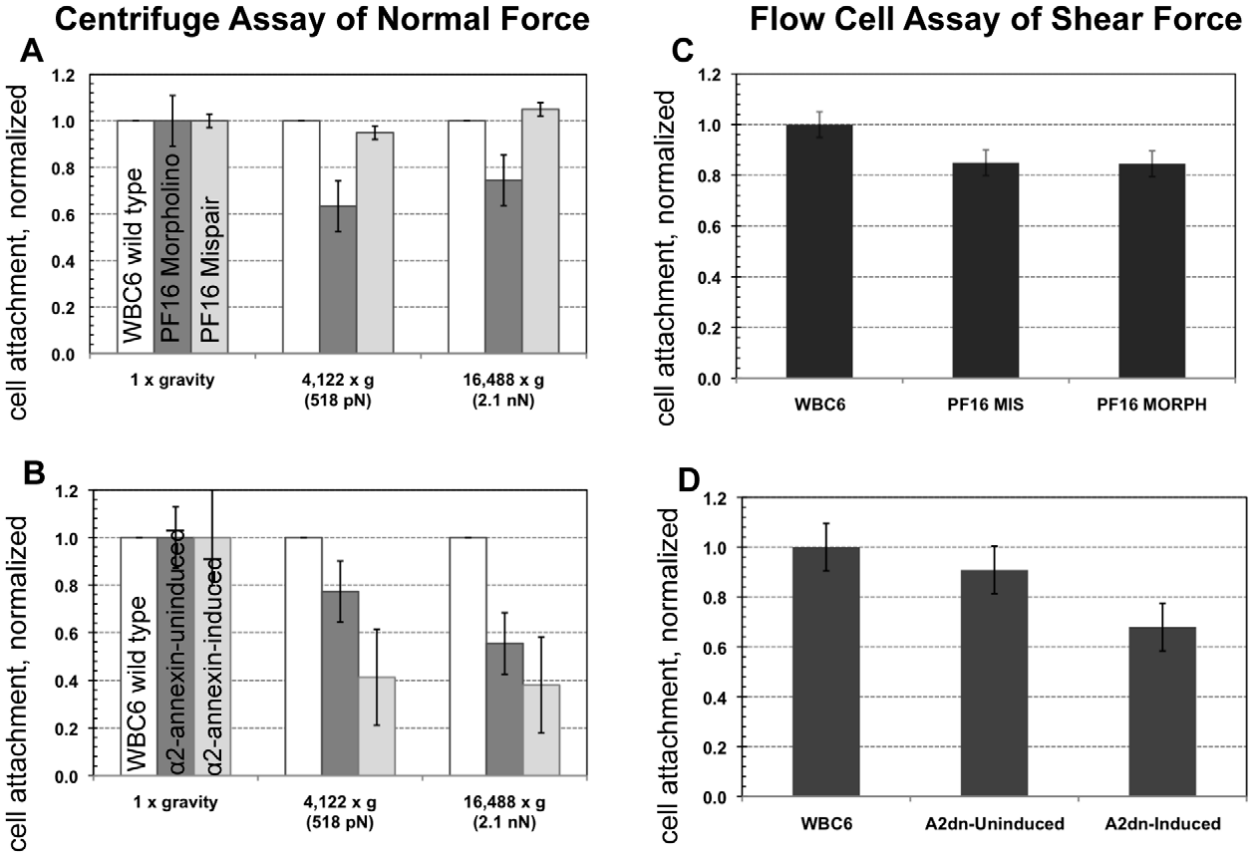
Trophozoites are able to maintain attachment when challenged with normal and shear forces, despite reduced flagellar beat frequency or diminished flagellar amplitude. The ability of cells to resist detachment was measured using both a centrifuge assay to calculate normal force and a flow cell assay to calculate shear force. Panel (**A**) shows the number of cells (n = 300, in triplicate) with reduced flagellar beat frequency that resist detachment after challenge, normalized to the number of non-centrifuged control cells (1x gravity) for each of two conditions (518 pN and 2.1 nN). Graph (**C**) shows the number of cells (n = 125, in triplicate) that resist detachment in a flow chamber when challenged with laminar flow (1.5 nN of force) normalized to control cells (no flow). Greater than 70% of the population was able to resist detachment under both types of force. The dominant negative alpha2-annexin (alpha2-annexin dn) strain was challenged under the same conditions of normal (**B**) (n = 4,000 in triplicate) and shear force (**D**) (n = 400 in triplicate). Despite diminished flagellar waveform amplitude and overexpression of a plasma membrane protein, 50% or more of the dominant negative alpha2-annexin::GFP (D122A, D275A) population was able to resist detachment.

**Figure 5:**
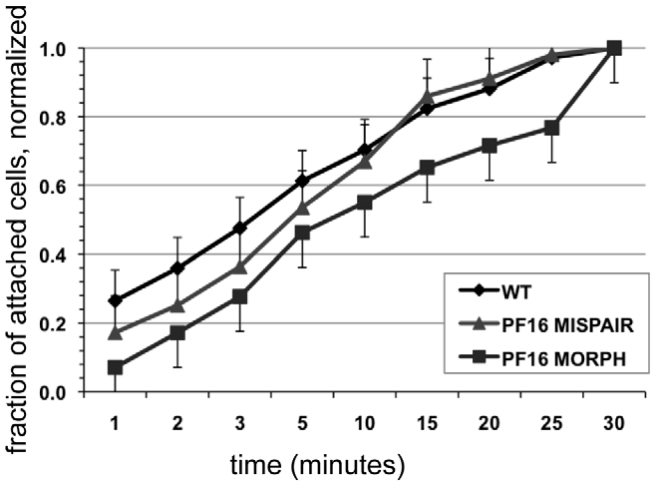
Defective flagellar beating hinders the initiation of attachment but does not affect the ability to maintain attachment. Attachment was imaged and quantified over thirty minutes in wild type, in the PF16 morpholino knockdown and in cells treated with mispair morpholino (see also Video S5). Cell counts of attached trophozoites were normalized to the total number of cells attached at thirty minutes. Error bars indicate standard error of the mean; n>100 in triplicate.

We were interested in attachment mechanics and the precise contribution of flagellar beating to attachment, either directly via hydrodynamic suction [7], [13], or indirectly via cellular positioning prior to attachment. We examined the role of ventral flagellar beating during the early stages (positioning) and later stages (maintenance) of attachment in live trophozoites. Using Total Internal Reflection Microscopy (TIRFM) of trophozoites labeled with a fluorescent plasma membrane dye, we defined distinct stages of attachment based on cellular and ventral disc contacts with the substrate surface (Figure 1). To test whether flagellar motility is required for giardial attachment, we used a morpholino-based knockdown [14] of the axonemal central pair protein PF16 to generate a strain with defects in flagellar beating. Knockdown of giardial PF16 resulted in various defects in all flagella, including defects in the rate of flagellar beat and/or flagellar length (Figure 2). Secondly, we constructed a strain with defects specific to the ventral flagellar waveform by overexpressing a dominant negative [15], [16] ventral flagella-specific alpha2-annexin (Figure 3). By assessing attachment in both types of trophozoites with defective flagellar motility, we demonstrate that defects in flagellar beating and coordination do not significantly affect attachment, with respect to disc contacts with the substrate surface or the ability to withstand normal forces and shear forces (Figure 4). Deficiencies in flagellar motility do, however, result in slower attachment during earlier stages when motility is required for positioning the ventral disc against the substrate surface (Figure 5). Thus, we propose that flagella contribute indirectly to attachment by positioning the cell, but ventral flagellar beating, specifically, is not directly involved in generating suction forces underneath the ventral disc.

## Results

The existence of channels for fluid flow is essential to the postulated hydrodynamic model of attachment [7]. Thus, to evaluate the hydrodynamic model of attachment, we first defined specific contacts of the trophozoite with the substrate surface. We then examined the role of flagellar motility during both the early (positioning) and maintenance stages of attachment by assaying the ability of giardial strains with defects in flagellar beating to initiate and maintain attachment as defined by these contacts. Because giardial attachment to surfaces is rapid and dynamic [17], [18], [19], the assays we use to quantify attachment are based upon live imaging.

### TIRFM characterizes surface contacts during distinct stages of attachment and detachment in live trophozoites

To investigate the direct or indirect contributions of flagellar beating to attachment, we defined the general stages of attachment and detachment based on cell body and ventral disc surface contacts using TIRFM of trophozoites stained with a plasma membrane dye (Figure 1, panels C-F). TIRFM uses an evanescent wave that penetrates only 100 nm into the sample medium, enabling selective visualization of surface regions of cells. Trophozoites first skim and contact the surface with the anterior section of the ventrolateral flange (Figure 1). Secondly, the perimeter of the ventral disc touches the surface, forming a continuous contact, or “seal” at the area of the lateral crest. The lateral shield then presses against the substrate, followed by the bare area region within the ventral disc. We noted bare area contact in 76% of attached cells (n = 97 cells). During maintained attachment, we observed a continual surface contact via the lateral crest seal, around the entire ventral disc. Giardial attachment to biological or inert surfaces is reversible and occurs within seconds [17], [18], [19]; here attachment to a glass substrate occurred in less than one to several seconds. In prior work using transmitted light contrast techniques this seal was postulated to be a channel [6], [7]. The lateral crest seal is the first indicator that the hydrodynamic model as described is invalid [6], [7].

Detachment begins with release of the bare area from the surface, followed by release of the lateral shield (Figure 1). The disc seal becomes discontinuous, specifically at the posterior lip, and finally the cell detaches to swim in the medium. When the cell skims (Video S1) the disc seal and ventrolateral flange remain in close contact with the substrate while the bare area region lifts. Bare area contact reappears when the cell pauses or reattaches. Ventral flagellar beating is close to the surface and thus readily imaged in TIRFM. The beating of other flagella can only be observed when those flagella are motile and come within 100 nm of the substrate.

In contrast to previous reports [7], we did not observe an arched groove between the disc perimeter and the ventrolateral flange (Figure 1). The ventrolateral flange does not have an arched profile and remains flat against the substrate, as does the lateral shield. Most notably, we did not observe a “Y”-shaped ventral channel present between the posterior lip of the ventral disc that continues into a ventral-caudal stem, postulated to conduct a hydrodynamic current [6], [7]. In contrast, we observed a complete and continuous disc seal (Figure 1). The surface contacts observed using TIRFM are consistent with ultrastructural SEM and TEM images of attached cells as well as interference-reflection microscopy [20].

We measured a wild type ventral flagellar mean beat frequency of 9 Hz with a synchronous waveform beat from base to tip along the longitudinal axis of the cell, similar to previous reports [10]. In contrast to prior work that used trophozoites scraped from mouse intestine [6], [7], we determined the mean amplitude of the ventral flagellar waveform to be 2.04 µm. Synchronous ventral flagellar beating was observed once the trophozoite made a seal with the lateral crest (Figure 1 and Video S1). Notably, a change in frequency and amplitude during skimming and swimming correlated with changes in the directional motility of the cell, as previously reported [6], [7].

### Morpholino knockdown of the central pair protein PF16 results in defects in flagellar beating

PF16 was first characterized in the green alga *Chlamydomonas reinhardtii* as a highly conserved armadillo-repeat protein localizing to the C1 microtubule of motile flagella [21], [22], and required for proper flagellar waveforms and motility [23], [24], [25]. We expected the *Giardia* PF16 homolog to have a similar function in generating proper waveforms and motility in the eight giardial flagella. To test whether ventral flagellar beating is necessary to create hydrodynamic flow for attachment, we used a PF16-specific morpholino (see Methods and [14]) to block translation of the protein. The anti-PF16 morpholino knockdown was confirmed via Western blot and immunostaining (see Figure S1) using an integrated *PF16*::HA strain [26].

As with other flagellates [21], [22], [27], the knockdown of PF16 resulted in significant flagellar motility defects. Twenty-four hours after morpholino electroporation, the anti-PF16 treated trophozoites exhibited erratic behavior in all flagella. Both the wild type and the PF16 mispair control had ventral flagella of similar lengths (14.4 µm for the membrane-bound portion, measured from the flagellar exit point at cell body to the flagellar tip), a sigmoidal ventral flagellar beat pattern, and similar beat frequencies (9 Hz) and amplitudes (2.04 µm) (Figure 2 and Video S2). The anti-PF16 morpholino-treated cells sustained amplitudes similar to wild type but displayed an erratic flagellar beat. Twenty-four hours after anti-PF16 morpholino introduction, 71% of cells exhibited a significantly decreased ventral flagellar beat (mean = 4 Hz) with 200 millisecond pauses (Figure 2).

Flagellar motility is also thought to be required for later stages of cytokinesis in *Giardia* [28]. Forty-eight hours after anti-PF16 knockdown, many cells in the population lagged in their ability to complete cytokinesis so that many daughter cells remained connected via their posterior cell bodies. Total paralysis was not observed, but ventral flagellar beating slowed to 2.3 Hz, a significant reduction from 9 Hz found in wild type (n = 50). We also observed that the membrane-bound regions of the ventral and caudal flagella were one quarter and one third, shorter, respectively (Figure 2, Video S2). A significant number of cells also exhibited dorsal flexion paralysis which could result in detachment, thus this time point was not included in the cell attachment assays.

### Trophozoites treated with anti-PF16 morpholino can maintain attachment despite flagellar beating defects

Using TIRFM with live imaging, we investigated the ability of the anti-PF16 morpholino transformant with flagellar beating defects to form proper surface contacts on glass coverslips (see Figure 2). Despite the significant defects in flagellar beat rate (Figure 2 and Video S2), trophozoite surface contacts in anti-PF16 morpholino-treated cells were similar to wild type (Figure 1). We observed no defects in the lateral body contacts, bare area contacts or the continuity of the disc perimeter seal.

To assay the ability of the PF16-knockdown trophozoites with defective flagellar beating to maintain attachment, we next challenged live morpholino-treated trophozoites with two biophysical assays. First, using a centrifuge assay of increasing normal forces, we noted that the anti-PF16 knockdown population maintains attachment against normal centrifugal forces up to 2.1 nN (Figure 4) similar to wild type trophozoites [21]. To assay the ability to withstand shear forces, we used a flow cell assay [20]. The anti-PF16 morpholino knockdown trophozoites were able to maintain attachment when challenged with 1.5 nN of shear force, equal to the mispair morpholino control (Figure 4). The prediction of the hydrodynamic model is that trophozoites would detach once the flagellar beat decreased, and thus would be incapable of maintaining steady state attachment when challenged with force. Despite the fact that the ventral flagella beat erratically and were noticeably shortened in length (see above), the PF16 knockdown trophozoites retained the ability to initiate and maintain attachment comparable to wild type trophozoites.

### Overexpression of a dominant negative alpha2-annexin (D122A, D275A) results in defects in the ventral flagellar waveform

Annexins are membrane-scaffold proteins that generally link the cytoskeleton to the periphery of negatively charged, acidic phospholipid membranes in a Ca^+2^-regulated manner [29]. Several annexin homologs have been shown to localize specifically to various pairs of flagella [30]. Alpha2-annexin was previously shown to localize to the ventral flagella [30]; thus, dominant negative annexins could specifically inactivate the waveform of the ventral flagella. We confirmed the localization of alpha2-annexin to the ventral flagella (Figure 3) in both live and fixed cells using a GFP tag [30]. Alpha2-annexin::GFP localizes to 87% of the cell population, strongly to the ventral flagella (signal intensity mean = 1650), and to a lesser degree, the plasma membrane of the ventral disc (signal intensity mean = 700) and the cell. We observed that the ventral flagellar waveform, synchrony, beat rate and frequency in the alpha2-annexin::GFP strain equaled that of the WBC6 wild type strain. We measured a negligible decrease in amplitude at 1.71 µm, as compared to 2.04 µm in wild type, yet we observed no growth or attachment defects in the alpha2-annexin::GFP strain.

To test the particular role of ventral flagellar beating in attachment, we created a strain with defects in the ventral flagellar waveform caused by overexpression of a tetracycline-inducible dominant-negative alpha-2 annexin. Specifically, we modified amino acid residues in two of four high-affinity calcium-binding domains in the giardial alpha-2 annexin from asparagine to alanine (D175A, D275A), which has previously been shown to generate dominant negative annexins [31] (Figure S1). We observed and quantified significant defects in 82% of the alpha2-annexin (D175A, D275A)::GFP population, specifically in the amplitude of the ventral flagella waveform (as compared to wild type (reviewed recently in [9]) at both 24 and 48 hours after induction of the alpha2-annexin dominant negative construct. Specifically, the amplitude of the ventral flagellar waveform was significantly decreased from 2.04 µm in wild type to 0.85 µm in the dominant negative strain. A C-terminal GFP tag allowed visualization of the dominant negative alpha2-annexin::GFP, which localized to the ventral flagella plasma membrane (Figure 3) and somewhat to the ventral disc, albeit with a weaker signal than the alpha2-annexin::GFP strain. Two of the four active calcium-dependent binding sites were mutated, leaving only two with the ability to bind the membrane, likely resulting in lower signal intensity.

### The dominant negative alpha2-annexin (D122A, D275A) strain has a decreased ventral flagellar waveform but minimal defects in attachment

Because overexpression of the dominant negative alpha2-annexin resulted in defects of the ventral flagellar waveform, we assessed the ability of this strain to attach using both TIRFM and live biophysical assays of normal [19] and shear forces. Because prolonged exposure to fluorescence microscopy can induce changes in flagellar beating, we limited our observations to less than 30 minutes in temperature-controlled, closed environments. Flagellar beat measurements were captured with TIRFM at very low (10 ms) exposures for two seconds, and then confirmed with phase contrast microscopy. Trophozoites overexpressing the dominant negative alpha2-annexin could still form a seal at the ventral disc perimeter (Figure 3), and could resist increased normal and shear forces despite the observed defects in ventral flagellar waveform (Figure 4). We did observe a decrease in the ability of the dominant negative alpha2-annexin strain to withstand normal forces in the centrifuge assay as compared to wild type that could be attributed to an increased rigidity in the plasma membrane of the ventral disc as well as the ventral flagella.

### The rate of attachment slows after treatment with anti-PF16 morpholino

Rather than being directly involved in generating a hydrodynamic current, flagellar motility could be important for positioning trophozoites so that the ventral disc is oriented parallel to the substrate. Defects in cellular positioning would not necessarily affect the overall number of cells attached but could slow the initial rate at which trophozoites attach to surfaces. Using live imaging we observed that the anti-PF16 morpholino-treated trophozoites often settled near the substrate, yet were oriented incorrectly with the disc side facing away from the surface. We then used time-lapse imaging of live trophozoites attaching to the bottom of a 96-well plastic cell culture plate and quantified the number of cells able to attach at specific intervals over a thirty-minute period (Figure 5). As compared to wild type and mispair morpholino controls, the anti-PF16 morpholino cells took longer to attach to the substrate (Figure 5) and had a decreased skimming motility as compared to wild type. Moreover, the rate of attachment was significantly decreased at each time point over a thirty-minute period in the trophozoites with defective flagellar beating.

## Discussion

In the highly variable environment of the small intestine, *Giardia* trophozoites need to remain attached to the intestinal villi to proliferate and to avoid peristalsis. Proposed models of giardial attachment to surfaces can be broadly categorized as: ligand-independent interactions (electrostatic or van der Waals) [19], ligand-specific interactions [32], [33], [34], [35], [36], “clutching mechanisms” [6], [17], [32], [37], [38], or “suction”-mediated mechanisms [6], [7], [17], [18], [19], [37], [38], (reviewed in [37]). The majority of the proposed models, particularly those involving flagellar motility, are primarily based on microscopic observations [7], [38], [39], [40], [41], [42], [43], [44], [45]. Understanding the active or passive contribution of the flagella to attachment dynamics is of fundamental importance toward developing new classes of anti-giardial compounds.

### Surface contacts of the ventral disc and cell body define distinct stages in attachment and detachment

Trophozoites attach to both biological substrates such as the intestinal microvilli (*in vivo* attachment) and inert substrates such as plastic or glass (*in vitro* attachment); however, the precise contacts of the ventral disc or the trophozoite cell body with the surface have not been defined. Giardial attachment has been broadly defined as the number of cells that remain adhered to a given surface after an experimental treatment [33], [36], [41], [46], [47], [48]. Based on these definitions, attachment has been quantified using three types of experimental approaches: 1) direct/indirect counts of attached and unattached cells [33], [36], [41], [46], [47], [48]; 2) live imaging [7], [17], [18], [38], [49], [50]; and more recently 3) a novel centrifuge assay of normal attachment force [19]. Most attachment assays have counted the number of adherent trophozoites at the population level after long incubation periods (∼2-24 hours), as opposed to quantifying the attachment dynamics of individual trophozoites under physiological conditions comparable to the host. Attachment generally has not been correlated with cell viability despite the common understanding that *Giardia* detaches when dividing [28], [45], when non-viable, or when exposed to oxygen or low temperature [46].

Using TIRFM to capture attaching trophozoites (Figure 1), we demonstrate that a binary (on/off) conception of attachment is misleading and overly simplistic. Giardial attachment occurs as a stepwise process proceeding in degrees of cellular contact with the surface (Figure 1). Four stages of attachment include skimming, disc seal formation (via the lateral crest), lateral shield contact and bare area contact (Figure 1). In each stage the disc remains concave, with only the disc edges and later the bare area contacting the surface. While the timing of these stages can vary from less than one second to several seconds, the stages of surface contacts during attachment and detachment (Figure 1) always occur in this stepwise fashion. Quantifying cell surface contacts also permits the assessment of attachment defects resulting from drugs or potential molecular genetic disruptions of the attachment mechanism.

During detachment, the disruption of surface contacts of the cell body and ventral disc occur in reverse order to the stages of attachment (Figure 1). Movements of the caudal pair of flagella are thought to generate the flexing of the posterior trophozoite “tail” region, indirectly resulting in detachment [10]. Our TIRFM analysis indicates that the tail region does not flex toward the surface prior to detachment and thus do not support this notion. Nonetheless, whether lateral tail flexion or dorsal tail abduction causes detachment still needs to be directly tested.

Our analysis of surface contacts also has specific implications for giardial attachment models (summarized recently in [8]). Soloviev and Holberton [7] proposed that a hydrodynamic force generated by ventral flagellar beating created a negative pressure differential under the adjacent disc to cause suction (also see Figure 1). The ventral flagella would theoretically create a fluid flow transmitted through a ventral disc channel toward a posterior disc cavity (Figure 1B). Thus, hydrodynamic-based suction would be contingent upon the presence of an “arched profile” of the ventrolateral flange, a ventrolateral channel around the perimeter of the disc and a hypothetical “disc portal at the posterior rim of the disc” [51]. Trophozoite surface contacts using TIRFM (Figure 1C-F) demonstrate that the disc perimeter forms a continuous seal with the surface. Further, we do not observe either an anterior or a posterior channel when cells are attached. What was previously considered to be a putative channel is, in fact, the lateral crest of the disc pressed against the substrate to form the seal. The anterior portion of the cell, including the ventrolateral flange, may be a flexible region.

### Overall defects in flagellar motility or specific defects in ventral flagellar beating do not adversely affect attachment

We used two molecular genetic approaches to generate trophozoites with flagellar motility defects to test further whether the ventral (or any) flagellar beating is necessary for giardial attachment.

First, we generated general flagellar beating defects by knocking down the giardial homolog of PF16 (Figure 2), a component of the central pair apparatus of axonemes [27] that localizes to the C1 microtubule of motile (“9+2”) flagella. In *Chlamydomonas*, mutations in *pf16* result in paralyzed flagella [22], and in trypanosomes RNAi of *pf16* results in erratic flagellar twitching [21], [25]. PF16 knockdown can result in axonemal ultrastructural defects, paralyzed flagella, or poorly beating flagella and can ultimately result in axonemes lacking the C1 microtubule [21]. Knockdown of PF16 in *Giardia* caused a significant decrease in flagellar beat frequency (Figure 2) yet did not cause complete paralysis of flagellar motility. Transient paralysis or pausing did occur every six to eight beat cycles. We also observed shortened ventral and caudal flagella, with one caudal flagellum consistently shorter than the other (Figure 2). This may be a preliminary indication that one caudal flagellum is older than the other caudal flagellum. Alternatively this observation supports findings in *Chlamydomonas* that indicate that when a new flagellar axoneme is under construction, length regulation is not limited to the new flagellum, but affects the pair as a whole [52].

To generate ventral flagellar beating defects specifically, we created and overexpressed a dominant negative version of the alpha2-annexin in trophozoites (Figure 3 and Figure S1). Based on the observed ventral flagellar defects, alpha2-annexin is a presumptive component of the ventral flagellar membrane scaffold. Annexins mediate interactions between the cytoskeleton and the plasma membrane [31], [53], and in *Giardia*, the flagella-specific annexins may regulate the stabilization of flagellar membranes by linking axonemal microtubules to the plasma membrane [54]. We show that parasites are still able to maintain proper surface contacts, despite a ventral flagella waveform of less than half that of wild type due to the overexpression of the dominant negative alpha2-annexin. The fraction of cells able to maintain attachment under normal and shear forces was slightly reduced but because the alpha2-annexin protein localizes to the plasma membrane of the ventral disc as well as the flagella, we surmise that increased ventral disc membrane rigidity may affect the disc attachment dynamics. Eighty-two percent of the trophozoites exhibit the defective flagellar motility phenotype. If ventral flagellar motility were essential for attachment as predicted by the hydrodynamic model [7], only 18% of the cells would be expected to remain attached due to the lack of penetrance of the phenotype. We observed that over two-thirds of parasites remained attached when compared to the uninduced construct (Figure 4); there is not a statistical difference between these percentages. Despite this slight reduction, the formation of proper surface contacts (as visualized by TIRFM of individual cells with impaired motility (Figure 3)) supports the argument that ventral flagellar beating is not directly responsible for attachment.

Thus, both the PF16 knockdown and the overexpressed dominant negative, ventral flagella-specific annexin strain could initiate and maintain attachment, as measured by the degree of surface contact (Figures 2 and 3) or in live imaging-based biophysical assays (Figure 4). The ability of attached cells to resist shear and normal forces, despite a decreased waveform amplitude or flagellar beat rate indicates that maintenance of attachment is independent of fluctuations in flagellar motility [7], and does not support the hydrodynamic model. While ventral flagellar beating is coincident with attachment, ventral flagellar beating neither directly causes nor directly results from the process of attachment.

### Flagellar beating is critical for cellular orientation and positioning prior to attachment

Trophozoites colonize the small intestine after excystation, and flagellar motility is likely required for orientation and positioning the trophozoite against the intestinal villi and for resisting peristaltic currents. Therefore, independent of creating a hydrodynamic current, flagellar motility could have an indirect role in positioning and orienting trophozoites with the ventral disc parallel to *in vivo* or *in vitro* surfaces prior to attachment.

Overall deficits in flagellar motility should affect both rotational motility (via the anterior flagella) and skimming motility (via the ventral flagella). Over time, trophozoites with aberrant motility may settle and attach to surfaces, but the time required for orientation and positioning prior to attachment could be significantly longer. In support of this idea, the anti-PF16 morpholino knockdown, with universal defects in flagellar motility and/or length (Figure 2), did take significantly longer to attach at each time point over thirty minutes during time-lapse imaging (Figure 5), and often settled to the substrate with the ventral disc up or remained swimming near the substrate (Video S4).

Anterior flagellar motility is proposed to be responsible for rotational movements [10], thus anterior flagellar defects resulting from the PF16 knockdown would affect trophozoite orientation.

Beating of the ventral flagella has been proposed to generate forward movement [10], and thus the disruption of ventral flagellar function (Figure 3) contributed to the inability of trophozoites to efficiently skim. Skimming motility allows the trophozoite to remain close to the substrate while searching for a desirable attachment location. Temporal lags in attachment due to flagellar motility defects might even result in more significant decreases in attachment *in vivo* due to consistent peristaltic flow.

### Flagellar motility indirectly contributes to giardial attachment

Models of giardial attachment are not mutually exclusive, and it is clear that site recognition, flagellar motility, and disc-mediated suction each contribute to *in vivo* attachment. With respect to site recognition, ligand-specific interactions could be involved in the parasite's selective colonization of the small intestine [32], [33], [34], [35], [36] in conjunction with flagellar motility. Despite a suggested role for sugars or lectins in mediating specific interactions of *Giardia* with host cells *in vivo* (reviewed in [55]), lectin-mediated site recognition is not necessary for attachment *in vitro*.

Once a site is recognized in the host, and flagellar motility positions the trophozoite, attachment may occur directly via a suction-based mechanism; suction is reported to be sufficient for *in vitro* attachment [19]. In the absence of a hydrodynamic current created by ventral flagellar beating to generate a negative pressure differential or suction underneath the disc, we propose that suction could be generated directly via a conformational change of the ventral disc. In this model, the lateral crest would first initiate the disc “seal” as observed in TIRFM (Figure 1). Next, a negative pressure differential would occur under the ventral disc via conformational changes of principal disc structures (MTs, microribbons, crossbridges and/or motor proteins). These structures would then relax back to their original conformation, producing a pressure differential between the arched disc and the substrate, consistent with TEM studies [11]. Alternatively, the ventral disc of the trophozoite could undergo a conformation change via protrusion of the bare area (as seen in the TIRFM, Figure 1). This change in the ventral disc volume would result in decreased fluid pressure due to the displaced fluid. The low pressure under the disc, compared to the surrounding high pressure of the environment would result in a pressure differential that may explain *Giardia'*s mode of suction-based attachment.

Flagellar motility prior to attachment is a key factor in *Giardia*'s pathogenesis and colonization of the host small intestine. The work presented here underscores that flagellar motility is important for positioning and orienting trophozoites prior to attachment. The consequence of inhibition of flagellar motility is a decrease in number of attached cells *in vivo* as is apparent *in vitro* (Figure 5). Thus, drugs affecting flagellar motility could indirectly result in lower levels of attachment by limiting the number of cells that can position the ventral disc properly against a surface and against peristaltic flow.

## Methods

### Strains and culture conditions

*G. intestinalis* strain WBC6 (ATCC 50803) trophozoites were maintained in culture at 37°C in modified TYI-S-33 medium with bovine bile [56] in sterile 13 ml screw-capped disposable tubes (BD Falcon) and incubated upright without shaking. For imaging, trophozoites were also grown on coverslips placed in 8-well dishes in a sealed chamber (PlasLabs) and gassed with 100% N_2_ to maintain a low oxygen atmosphere. The chamber was incubated at 37°C prior to live cell imaging.

### Construction of the C-terminal *pf16*-3HA integrated strain

Integration of an HA-tagged version of the *pf16* gene permitted the assessment of morpholino knockdown in the absence of a specific anti-PF16 antibody. The C-terminal portion of the PF16 gene was cloned in frame to a 3HA-tag and then into a pJET vector containing a neomycin selectable marker as previously described [26]. *Nru*I was used to linearize the vector. Trophozoites were transformed with linearized vector by electroporation as previously described [26], resulting in C-terminal fusion of a 3HA tag to at least one endogenous copy of the PF16 gene. Transformants were selected with 200 µg/ml neomycin G418 (Sigma). Endogenous integration of the construct was confirmed using PCR primers specific to the N terminus of the *pf16* gene and the 3XHA epitope tag. The localization of PF16 to all eight axonemes was verified with immunostaining using a monoclonal anti-HA antibody (Sigma H9658) at a 1∶100 dilution and an Alexa 594 goat anti-mouse IgG secondary antibody (Invitrogen) at a 1∶200 dilution.

### Morpholino-based knockdown of the central pair protein PF16

To knock down the giardial axonemal central pair PF16 homolog (GiardiaDB GL50803_16202), anti-sense morpholino oligonucleotides (GeneTools) were designed to the 5′ flanking region and first codons [14] with the following sequence: 5′ TACGACGAAGCGATTAGTTGCCATG 3′. Anti-PF16 morpholino oligonucleotides (100 µM final concentration) were electroporated into log phase trophozoites as previously described [14]. Morpholino-transformed cells were then incubated for 24 and 48 hours before the phenotype was assessed. To control for off-target effects of the electroporation or of the electroporated morpholino oligonucleotides, both sterile water or a morpholino with five mismatches (5′ TATGACAAAGCGGTTAGTAGCCATA 3′) were also electroporated. Following electroporation of PF16 specific morpholino oligonucleotides or controls, cell morphology, flagellar beating, and attachment were assessed in both live and fixed trophozoites (see below).

To determine the extent of knockdown, Western blotting was used to assay the HA-tagged PF16 levels in crude preparations of wild type *G. intestinalis* WBC6 or in extract from the integrated HA-tagged PF16 strain. *Giardia* PF16-3HA protein was detected using a 1∶2000 dilution of anti-HA antibody (mouse monoclonal, Sigma H9658) and an HRP-conjugated secondary antibody (Bio-Rad) at 1∶4000 dilution. The blot was also probed with anti-actin antibody at 1∶1500 to verify equal loading. The degree of PF16 knockdown was quantified using the Alpha Innotech Gel imaging and documentation system (Cell Biosciences).

### Construction of C-terminal GFP-tagged alpha-2 annexin::GFP and dominant negative alpha2-annexin::GFP (D122A, D275A) strains

By adapting a methodology that has been used to create dominant negative mutant forms of kinesin or dynamin in *Giardia* [15], [16], we constructed dominant negative mutations in the alpha2-annexin gene (D122A, D275A). We expected ventral flagellar defects with overexpression of alpha-2 annexin as it was previously shown to localize to the membrane-bound portions of the ventral flagella [30]. The conserved asparagines (D) were changed to alanines (A), as has been done previously for human annexins [29]. Alpha2-annexin :GFP fusions containing dominant negative mutations were placed under the control of a tetracycline-inducible promoter and the mutant protein was overexpressed in *Giardia* [57]. GFP tagging permitted the identification and characterization of trophozoites with significant levels of overexpression. Using the plasmid pTetGFPC.pac [15], we first created the tetracycline-inducible C-terminal alpha2-annexin::GFP fusion vector, pTetA2::GFPC.pac. We PCR amplified the alpha2-annexin gene (GiardiaDB GL50803_7796) using *Giardia* genomic DNA as a template with the following oligonucleotide primers:

TA2giaF: 5′ GATCAGGCGCGCCATGCCGAAGCTATCCCAGATCGTCGC 3′

TA2giaR: 5′ ACCGGTAGAGCGCCGGCTCCGGCTCCGGCCGCTGCGCCCTCCCTTAGGCGCCAGAGGGTACAGAG 3′

The PCR amplicon yielded alpha2-annexin flanked by 5′ *Asc*I and 3′ *Age*I restriction sites, permitting subcloning into pTetGFPC.pac. *G. intestinalis* strain WBC6 was transformed by electroporation with roughly 50 µg of pTetA2::GFPC.pac DNA using the GenePulserXL (BioRad) as previously described [58] with the following modifications: 375V, 1000 µF, 25 ohms. Episomes in transformants were maintained by antibiotic selection using 50 µg/ml puromycin (Sigma) [58].^.^

To create the pTetA2_D122A_D275A::GFP.pac dominant negative we used site-directed mutagenesis (Stratagene Quik-Change Site-Directed Mutagenesis Kit) with pTetA2::GFPC.pac as a template and the following PCR primers: For D122A: A2g122F: 5′ TTCATGAAGGCTGTCGGCCG 3′; A2g122R: CGGCCGACAGCCTTCATGAA 3′; and for D275A: A2g275F: 5′ GGTGCTTTGCTAAGCGCA 3′; A2g275R: TGCGCTTAGCAAAGCACC. The two point mutations (D122A and D275A) were created within the alpha2-annexin gene contained in the pTetA2::GFPC.pac construct; point mutations were confirmed by DNA sequencing. Constructs were electroporated into *Giardia* as described above. Induction of expression of alpha2-annexin in inducible strains was achieved by using 15 µg/ml of doxycycline per 12 ml culture for 24-48 hours. The maximal induction of transgenes occurred at 6-8 hours, and continued for over 48 hours after removal of doxycycline (see Video S1).

Induction and overexpression of alpha2-annexin (D122A, D275A) was confirmed using RT-PCR. Total cellular RNA was isolated from uninduced cells and from induced alpha2-annexin (D122A, D275A) cells at 24 and 48 hours after induction using the Cells-to-cDNA kit (Ambion). GFP overexpression was compared using the relative method of quantification [59], and GFP expression levels were normalized to the single copy giardial actin gene. Overexpression was determined from comparisons of normalized GFP expression in induced time points to uninduced controls. Thus, for quantitative analysis of expression, 1 µl aliquots of the cDNA synthesis reactions were used in subsequent actin (actF 5′ CCTGAGGCCCCCGTGAATGTGGTGG 3′ and actR 5′ GCCTCTGCGGCTCCTCCGGAGG 3′) and GFP-specific (GFPF 5′ GAGCTGTTCACCGGGGTGGTGCCC 3′ and GFPR 5′ CGGGCATGGCGGACTTGAAGAAGTCGTGC 3′) PCR amplifications with DyNamo HS SYBR Green qPCR Master Mix (Finnzymes). QPCR was performed with the Opticon 2 system (Bio-Rad). To demonstrate that RNA samples were not contaminated with DNA, control cDNA synthesis reactions were performed in the absence of reverse transcriptase.

### Immunostaining, light microscopy and image data analysis

Immunostaining and paraformaldehyde fixation of the alpha2-annexin::GFP and PF16::HA strains was performed as previously described [28] with anti-TAT1 tubulin (a kind gift from Keith Gull's laboratory) or anti-HA (Sigma) antibodies at 1∶100 with Alexa 594 secondary antibody at 1∶400 (Invitrogen). Images were collected with Metamorph image acquisition software (MDS Technologies) using a Leica DMI 6000 wide-field inverted fluorescence microscope with a PlanApo 100X, NA 1.40 oil immersion objective and captured with a Q imaging Rolera-MGi EMCCD. Serial sections were acquired at 0.2 µm intervals, and deconvolved using Huygens Professional deconvolution software (SVI). For presentation purposes, 2D maximum intensity projections were created from the 3D data sets. Simple histogram adjustments were made to increase visualization of the dominant negative alpha2-annexin::GFP (D122A, D275A) strain.

### Live cell imaging of attachment and flagellar beating using Total Interference Reflection Microscopy (TIRFM) and Differential Interference Contrast (DIC)

TIRFM uses evanescent waves that selectively illuminate and excite fluorophores in restricted regions of the specimen adjacent to the glass-water interface. This evanescent field decays exponentially away from the source, penetrating only about 100 nm into the sample [60]. For TIRFM, trophozoites were resuspended in 1X HEPES Buffered Saline (HBS) and incubated on ice for 10 minutes. To stain cell membranes, trophozoites were incubated for an additional 5 minutes on ice with CellMask Orange (final concentration of 2 µg/ml; Invitrogen). Stained cells were concentrated by centrifugation (900 x g for 5 minutes) and resuspended in 500 µl of warmed 37°C 1X HBS prior to imaging. A simple imaging chamber was created by mounting a coverslip to a standard slide with parallel lines of double-sided adhesive tape to define an imaging chamber. Cells were loaded into the chamber using a wide-bore pipette, and the edges were sealed with melted VALAP (equal parts Vaseline, lanolin, and paraffin). This chamber provided a microoxic environment sufficient for short-term imaging experiments up to one hour. Live cell imaging was performed in a microscope stage incubator (OkoLab) at temperature of 35-37°C using a 515 nm laser with 10 ms exposures at 30-60 ms intervals for less than two seconds; no CCD gain was used. Images were collected with a QuantEM 512 SC EMCCD camera (Photometrics) on a 3i Marianas inverted spinning disk confocal microscope system. The TIRF angle was achieved with a 100X 1.46 NA oil immersion objective. Controls for the axial plane showed loss of signal/resolution past 200 nm, which was confirmed with axial confocal controls. Slidebook software (Intelligent Imaging Innovations) was used for minor image processing such as cropping and 2D intensity plots.

### Live cell imaging and analysis of flagellar beat and motility

To assess flagellar beating and motility in live trophozoites, we used live imaging with DIC microscopy. Dead and/or unattached cells were decanted from culture tubes 1 to 4 hours prior to imaging, and fresh medium was added. The culture was then incubated on ice for 15 minutes, and pelleted by centrifugation at 900 X g at 4°C. Cell pellets were resuspended in 500 µl 37°C medium and transferred into a 35 mm glass bottom Petri dish (MatTek). The Petri dish was placed in a closed chamber and gassed with N_2_. The cells were allowed to attach to the dish for 1 hour before the dish was removed and the lid was sealed with Parafilm. This chamber provided a microoxic environment sufficient for short-term imaging of 1 to 4 hours.

Flagellar length and potential motility defects in the ventral flagellar pair (synchrony, waveform, and frequency) were assessed using live cell imaging. Flagellar length was measured for the membrane-bound portions from the flagellar pocket exit point to the distal flagellar tip. The synchrony of the ventral flagellar pair was observed by visually confirming whether ventral flagella beat in unison [6]. Ventral flagellar waveform was classified as sigmoidal (wild type) or abnormal and scored by the measurement of ventral flagellar amplitude and wavelength. Amplitude was measured by drawing a line from the basal bodies to the tip of the cell posterior and distance is measured from the line to the peak of the first wave [6] using Metamorph image acquisition software (MDS Technologies). Flagellar wavelength was measured by drawing a line from the first wave peak (proximal to the disc) to the next peak toward the tip. Ventral flagellar beat frequency has been previously reported to be 18 Hz [7], [10], [13]. Based on these measurements we satisfied proper Nyquist sampling by imaging at least twice the published frequency, thus capturing 36-45 images/second with 22-30 ms exposures.

### Quantification of shear forces of attachment using a laminar flow assay

The effect of shear forces on live trophozoites attached to a glass substrate was imaged using a syringe pump to create laminar flow with a temperature controlled Harvard Apparatus RC-31 parallel plate flow cell chamber (Warner Instruments) mounted onto an inverted Nikon Eclipse TS100 microscope with a 10X/0.25NA ADL objective and a Retiga 2000R CCD (Qimaging) as previously described [18]. The RC-31 chamber was fitted with a 100 µm narrow slot chamber gasket. The syringe pump was attached to the flow chamber via PE-10 and PE-90 tubing and a three-way stopcock for introduction of cells. For each time point, we chilled one 13 ml tube of giardial culture on ice and pelleted cells by centrifugation at 900 x g for 5 minutes. Cells were resuspended in 1 ml chilled medium and transferred into a 1 ml syringe. Cells were kept on ice no longer than 15 minutes.

Shear force experiments were performed on trophozoites 24 hours after the introduction of the PF16 morpholino or 24 hours after induction of the alpha2-annexin dominant negative construct. Specifically, the flow chamber was pre-warmed with 37°C 1X HBS for 5 minutes followed by warmed medium for 1 minute. A volume of 250 µl of trophozoites was introduced via a 1 ml syringe with an 18 gauge blunt needle into the chamber via a port on a three-way stopcock added to the media input line. Next, trophozoites were flowed into the flow cell chamber and allowed to attach for 5 minutes. The line was rinsed of floating cells until none were seen, at a rate of 0.5 ml/min. A pre-assay image was taken (Time = 0). Cells were then challenged with a 3 ml/min or greater laminar flow rate [18] for 1 to 3 minutes as images were captured via phase contrast at 10 second intervals. As a control for detachment, 5% bleach at 37°C was introduced into the chamber, whereby all cells detached within 5 seconds. The rate (greater than 3 ml/minute) and chamber area (208 mm) were then converted to a “shear” force of 1.5 nN. To quantify the fraction of cells that maintained attachment over a range of shear forces, cell counting was performed manually or using Metamorph image acquisition software (MDS Technologies). The proportion of attached morpholino-treated cells and/or alpha2-annexin dominant negative trophozoites were normalized to the wild type attached trophozoites.

### Assessing normal forces of attachment using a centrifuge assay

Defects in the normal forces of attachment in trophozoites were assayed at the population level using a physical attachment assay [19]. Briefly, PF16 morpholino or alpha2-annexin dominant negative trophozoites were cultured and pelleted as above. Cells were resuspended in 10 ml of chilled media, and then 3 ml were transferred to custom sample holders capped with thick, circular glass slides. The cells were incubated at 37°C for 1 hour in a microoxic chamber (see above) to allow attachment to the glass slides. Sample holders were centrifuged at 37°C in a hanging bucket centrifuge (Sorvall RC5C HB4 rotor 07) at 5,000 and 10,000 rpm (518 pN and 2.1 nN normal force). Non-centrifuged controls were prepared in the same manner and incubated at 37°C. Immediately after centrifugation, the glass slides were removed from the chamber and five fields (∼5000 cells) were imaged and counted in phase contrast (see above). The proportion of trophozoites maintaining attachment was normalized to the non-centrifuged control within that run (the number of attached cells following centrifugation was divided by the number of cells attached in the non-centrifuged sample to determine the fraction of cells detached).

### Live imaging and quantitation of pre-attachment dynamics

The ability of cells to initiate attachment was measured by assaying the number of cells attached in one microscopic field over a 30 minute time period. First, the medium in a 6 ml culture was exchanged with 1X HBS at pH 7.0 and incubated on ice for 10 minutes. To stain the cell membranes for cell counting, CellMask Orange (final concentration of 2 µg/ml; Invitrogen) was added, and the cells were incubated on ice for an additional 5 minutes. Stained cells were pelleted by centrifugation and resuspended in 250 µl of chilled 1X HBS (1.1×10^6^ cells per ml suspension concentration). For time-lapse imaging of attachment, 50 µl of stained cells were transferred to a microwell (Corning) overlaid with mineral oil and placed in a heated, closed stage chamber. The total number of attached trophozoites was imaged using a 10x/0.25 NA objective and quantified using time-lapse epifluorescence microscopy over a range of time intervals from 1 to 30 minutes. Total cell attached were counted using Metamorph image acquisition software.

### Accession numbers

Alpha2-annexin (XP_001706958; GiardiaDB: GL50803_7796), PF16 (XP_001705527: GiardiaDB: GF50805_16202.

## Supporting Information

Figure S1 **Immunostaining of integrated PF16::3HA tag and Western confirmation of anti-PF16 morpholino knockdown.** Panel A shows a Western blot of the integrated *pf16*::3HA strain, 24 hours after electroporation of: MilliQ water, PF16 mispair morpholino or anti-PF16 morpholino. *Giardia* actin was used as a loading control. The ratio under each column represents the amount of 3HA-integrated protein still present. These numbers indicate a block in translation of 15% due to electroporation, 29% due to introduction of morpholino (mispair control) and 65% due to the anti-PF16 morpholino. Panel B shows maximum intensity projections of fixed cells immunostained with anti-HA primary antibody and Alexa 594 secondary antibody. Twenty-four hours after knockdown, PF16 localizes to the cytoplasmic axonemes, as well as the membrane-bound portions of all flagella. The PF16 knockdown cells exhibit the same localization, in shortened flagella, with a 21% loss of fluorescence.
(TIFF)

Video S1 **Attachment and detachment of a wild type** *Giardia* **trophozoite, TIRFM movie.** Attachment is captured after a trophozoite skims along a warmed glass substrate. The ventrolateral flange maintains contact with the substrate while the cell is skimming and may be important in substrate recognition. In this example, the lateral crest also maintains close affinity with the substrate; however, the portion of lateral crest that makes contact during the skimming stage is variable. Once the cell begins to attach, a seal is formed with the lateral crest of the ventral disc. The lateral shield, on either side of the cell body, then presses against the substrate, quickly followed by a depression of the bare area plasma membrane. During detachment, the respective steps occur in reverse order. The bare area cell membrane disappears from view, and the posterior cell body and lateral shield lift up. This motion breaks the seal of the lateral crest and the cell proceeds to skimming using the ventrolateral flange.
(MP4)

Video S2 **Reduction in flagellar beat frequency resulting from anti-PF16 morpholino knockdown, DIC movie.** Movie A shows trophozoites 24 hours after electroporation with the PF16 mispair control morpholino. Like wild type, the ventral flagella have a regular, sigmoidal beat. Note that flagellar length is also similar to wild type (Figure 2). Movie B shows an extreme example of the PF16 phenotype, 48 hours after PF16 knockdown, where all flagella are significantly shortened. In Movie C, observe the shortened membrane-bound portion of the ventral flagella, likely representative of structures existing before translation of the PF16 protein was blocked. We also notice an increase in the surface area of the ventral flange, often including projections.
(MP4)

Video S3 **Diminished ventral flagellar beat frequency in the dominant negative alpha2-annexin::GFP (D122A, D275A) strain, DIC movie.** Movie A is the alpha2-annexin::GFP trophozoite exhibiting a typical sigmoidal ventral flagellar beat and amplitude. Movie B illustrates asynchronous beating of the ventral flagella, a phenotype detected in 12% of the dominant negative alpha2-annexin::GFP (D122A, D275A) trophozoites. Movie C shows the phenotype of the majority of cells, in which the amplitude of the ventral flagellar beat is significantly decreased.
(MP4)

Video S4 **Flagellar beating and lateral crest seal (wild type, alpha2-annexin dominant negative strain, anti-PF16 morpholino knockdown), TIRFM movie.** Movie A shows surface contacts made by a trophozoite during attachment to a glass substrate. Note the sigmoidal ventral flagellar beat and the continuous seal made by the lateral crest of the ventral disc. Movie B is an example of the dominant negative alpha2-annexin::GFP (D122A, D275A), exhibiting a decreased ventral flagellar beat amplitude, and Movie C is an example of the PF16 knockdown exhibiting normal cell movement but a decreased flagellar beat frequency. Movies B and C show that cells form surface contacts similar to wild type, including that of the lateral crest, despite disruption of ventral flagellar function.
(MP4)

Video S5 **Time-lapse movie of attachment (wild type, anti-PF16 morpholino knockdown, mispair control), epifluorescence movie.** Equal numbers of trophozoites, stained with CellMask Orange, were allowed to attach to a glass substrate in a warmed, anoxic environment. Wild type, PF16 mispair control and PF16 morpholino cells all attached with similar kinetics, but significantly fewer PF16 knockdown cells were able to initiate attachment at each time point (see Figure 5), indicating that the flagella are important for positioning the cell to the substrate.
(MP4)
