# Supplementary figures and images for: Giardia Flagellar Motility Is Not Directly Required to Maintain Attachment to Surfaces
Source: PLoS Pathog. 2011 Aug 4;7(8):e1002167. doi: 10.1371/journal.ppat.1002167 (PMC3150270; doi:10.1371/journal.ppat.1002167)

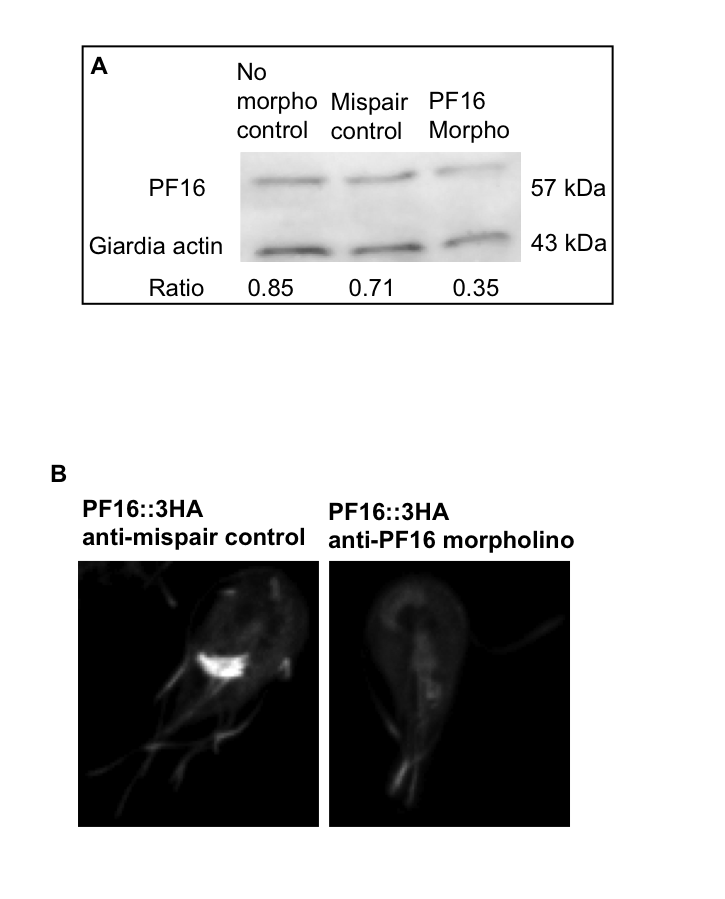

Supplement: Figure S1 — Immunostaining of integrated PF16::3HA tag and Western confirmation of anti-PF16 morpholino knockdown. Panel A shows a Western blot of the integrated pf16::3HA strain, 24 hours after electroporation of: MilliQ water, PF16 mispair morpholino or anti-PF16 morpholino. Giardia actin was used as a loading control. The ratio under each column represents the amount of 3HA-integrated protein still present. These numbers indicate a block in translation of 15% due to electroporation, 29% due to introduction of morpholino (mispair control) and 65% due to the anti-PF16 morpholino. Panel B shows maximum intensity projections of fixed cells immunostained with anti-HA primary antibody and Alexa 594 secondary antibody. Twenty-four hours after knockdown, PF16 localizes to the cytoplasmic axonemes, as well as the membrane-bound portions of all flagella. The PF16 knockdown cells exhibit the same localization, in shortened flagella, with a 21% loss of fluorescence. (TIFF) [file ppat.1002167.s001.tiff]
